# Vanadium toxicity in the thymic development

**DOI:** 10.18632/oncotarget.5798

**Published:** 2015-09-22

**Authors:** Wei Cui, Hongrui Guo, Hengmin Cui

**Affiliations:** ^1^ Key Laboratory of Animal Diseases and Environmental Hazards of Sichuan Province, Sichuan Agricultural University, Ya'an, China; ^2^ College of Veterinary Medicine, Sichuan Agricultural University, Ya'an, China

**Keywords:** vanadium, relative weight, cell cycle, apoptosis, protein expression, Immunology and Microbiology section, Immunity, Immune response

## Abstract

The purpose of this study was to define the toxic effects of vanadium on thymic development in broilers fed on diets supplemented with 0, 5, 15, 30, 45 and 60 mg/kg of vanadium for 42 days. We examined the changes of relative weight, cell cycle phase, apoptotic cells, and protein expression of Bcl-2, Bax, and caspase-3 in the thymus by the methods of flow cytometry, TUNEL (terminal-deoxynucleotidyl transferase mediated nick end labeling) and immunohistochemistry. The results showed that dietary high vanadium (30mg/kg, 45mg/kg and 60mg/kg) caused the toxic effects on thymic development, which was characterized by decreasing relative weight, increasing G_0_/G_1_ phase (a prolonged nondividing state), reducing S phase (DNA replication) and proliferating index (PI), and increasing percentages of apoptotic thymocytes. Concurrently, the protein expression levels of Bax and caspase-3 were increased, and protein expression levels of Bcl-2 were decreased. The thymic development suppression caused by dietary high vanadium further leads to inhibitive effects on T lymphocyte maturity and activity, and cellular immune function. The above-mentioned results provide new evidences for further understanding the vanadium immunotoxicity. In contrast, dietary 5 mg/kg vanadium promoted the thymic development by increasing relative weight, decreasing G_0_/G_1_ phase, increasing S phase and PI, and reducing percentages of apoptotic thymocytes when compared to the control group and high vanadium groups.

## INTRODUCTION

Vanadium, as a transition element or an important mineral distributed on earth, is used widely in the chemical industry and is contained in several foods, water particulates in air, fuel oils, and coal [1–4]. It is also considered to be a nutritionally essential trace element for animal species [5–8]. At present, vanadium or vanadium compounds have been known as new promising drugs for lowering blood glucose in diabetes due to their insulin-mimetic actions and ability to counteract insulin resistance [2, 4, 6, 9–20]. Also, they have been used in clinical practice to protect against tumor or cancer as a potent anticarcinogenic agent or antitumor drugs [4, 21–23]. At the same time, there have been a lots of reports focused on the toxic actions or side effects of vanadium and vanadium compounds, which constitutes another important area of research on vanadium.

The toxicity of vanadium compounds usually increases as the valence increases. The pentavalent compounds are the most toxic [24]. In vivo study in the laying hen has shown that dietary vanadium reduces body weight, feed consumption, egg production, egg weight and shell quality [25]. Oxidative stress or damage, reproductive toxicity (teratogenicity and embryotoxicity), organ and tissue injury, neurobehavioral injury or neurotoxicity, Mitochondrial dysfunction or injury, genotoxicity, cytotoxicity and blood toxicity induced by vanadium or vanadium compounds is well documented: *in vivo* and *in vitro* of both man and animals [4, 12, 18, 24, 26–52]. Sumanta et al. (2015) have reviewed that vanadium or vanadium compounds cause toxic effects including blood toxicity, abnormalities in development and reproduction (teratogenicity and embryotoxicity), neurobehavioral injury, morphological and functional lesions in liver, kidneys, bones, spleen and leukocytes, and inflammatory responses including rhinitis, pharyngitis, chronic productive cough, tracheobronchitis and bronchopneumonia [53]. Vanadium immunotoxicity is also appeared in the rat and rabbit [54]. Our previous studies have shown that dietary vanadium in 30 mg/kg and over causes lesions, oxidative damage, immunotoxicity, cytokine reduction, apoptosis, cell cycle arrest and microbiota alteration in the cecal tonsil, spleen, bursa of Fabricius, kidney, liver, intestine and serum [55–72]. Michael et al. (1987) have concluded that animals during periods of rapid growth are susceptible to vanadium toxicity, and increased lipid peroxidation may be one factor [73].

The thymus is the central lymphoid organ, and it forms and grows immediately after birth in response to postnatal antigenic stimulation and the demand for large numbers of mature T cells [74]. The number of mature T cells decides the biological function of the cells and is correlated to the cellular immune function of the body [75]. However, very limited data focus on the toxic effects of vanadium on thymic development in human beings and animals at present.

The aim of present research was to define the toxic effects of vanadium on thymic development by observing the changes of relative weight, cell cycle phase, percentages of apoptotic thymocytes and protein expression of apoptotic proteins (bcl-2, bax, and caspase-3) using the methods of flow cytometry, TUNEL (terminal-deoxynucleotidyl transferase mediated nick end labeling) and immunohistochemistry.

## RESULTS

### Clinical observation

Broilers grew much faster in 5 mg/kg group and much slower in 30, 45 and 60 mg/kg groups than in control group. Broilers in 45 and 60 mg/kg groups showed decreased feed intake and depression. Loss of body weight of 35.31%, 44.03%, and 68.05% was seen in 30, 45 and 60 mg/kg groups, respectively, at the end of the experiment when compared with that in the control group.

### Change of thymic relative weight

**Figure 1:**
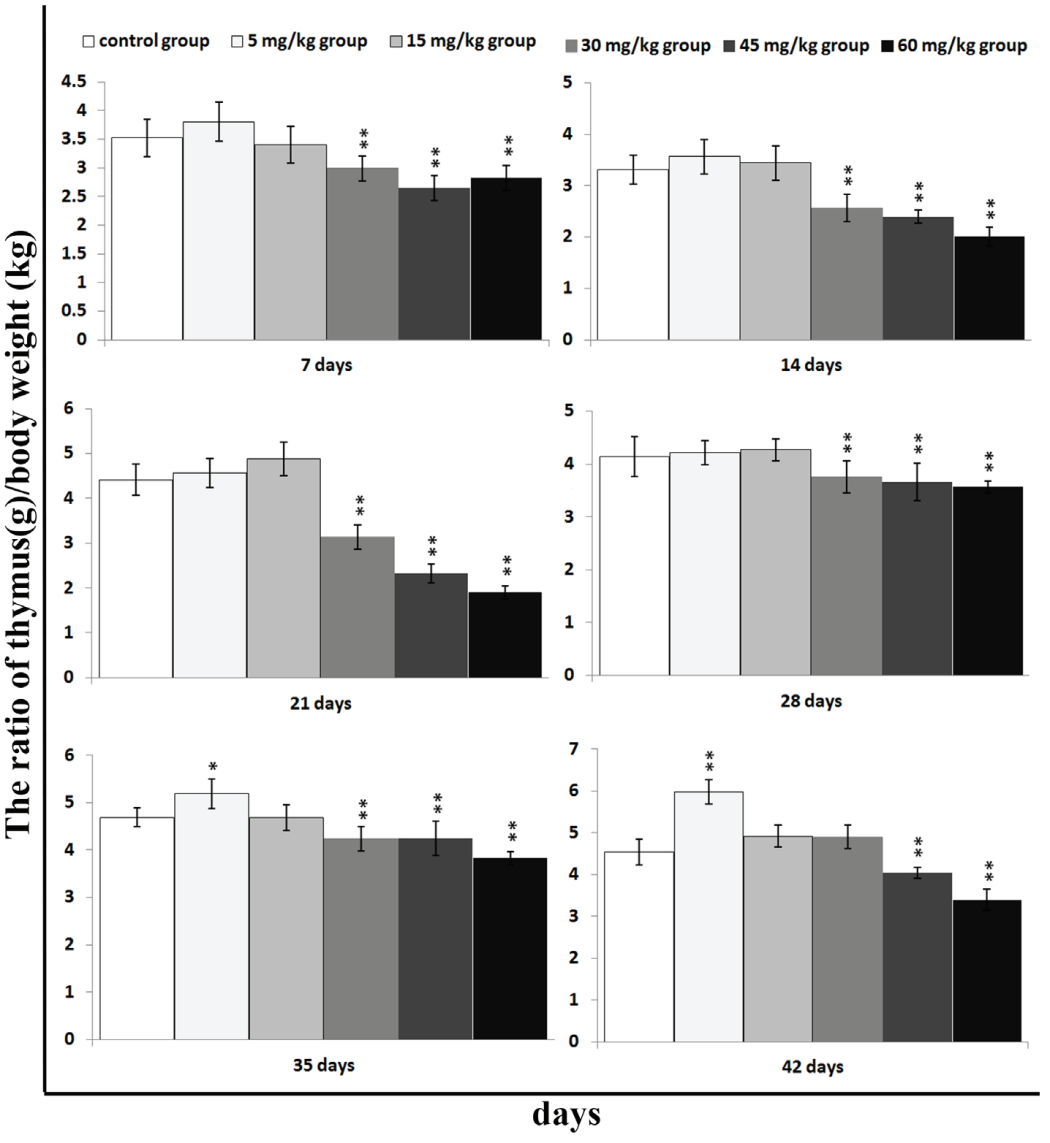
Changes of relative weight [thymus (g)/body weight (kg)] in the thymus Data are the means ± standard deviation (n=5) *p<0.05, compared with the control group **p<0.01, compared with the control group.

The thymic relative weight was significantly lower (*P* < 0.01) in the 30mg/kg, 45mg/kg and 60 mg/kg groups than those in the control group from 7 to 42 days of age, except 30mg/kg group at 42 days of age. At the same time, the thymic relative weight was significantly increased (*P* < 0.05 or *P* < 0.01) in the 5 mg/kg group at 35 and 42 days of age when compared with those in the control group. The results are shown in Figure 1.

### Changes of cell cycle in the thymus

The percentages of G_0_/G_1_ phase (a prolonged nondividing state) were significantly decreased (*P* < 0.05 or *P* < 0.01) in the 5 mg/kg group from 14 to 42 days of age. Moreover, the percentages of G_0_/G_1_ phase were markedly higher (*P* < 0.05 or *P* < 0.01) in 45 and 60 mg/kg groups than those in the control group from 14 to 42 days of age.

The percentages of G_2_+M phase were markedly increased (*P* < 0.05 or *P* < 0.01) in 5 mg/kg when compared with those in the control group. However, the percentages of the G_2_+M phase were lower (*P* < 0.05 or *P* < 0.01) in the 45mg/kg and 60mg/kg groups than those in the control group from 14 to 42 days of age.

The percentages of S phase (DNA replication) were significantly decreased (*P* < 0.01) in the 30mg/kg, 45mg/kg and 60mg/kg groups from 14 to 42 days of age, and were increased (*P* < 0.05) in the 5mg/kg group at 14 days of age when compared with those in the control group.

The proliferating index (PI) value was markedly increased (*P* < 0.05 or *P* < 0.01) in the 5 mg/kg from 14 to 42 days of age. Meanwhile, the PI was markedly lower (*P* < 0.05 or *P* < 0.01) in the 45mg/kg and 60 mg/kg groups from 14 to 42 days of age and in the 30 mg/kg group at 42 days of age than that in the control group.

**Figure 2:**
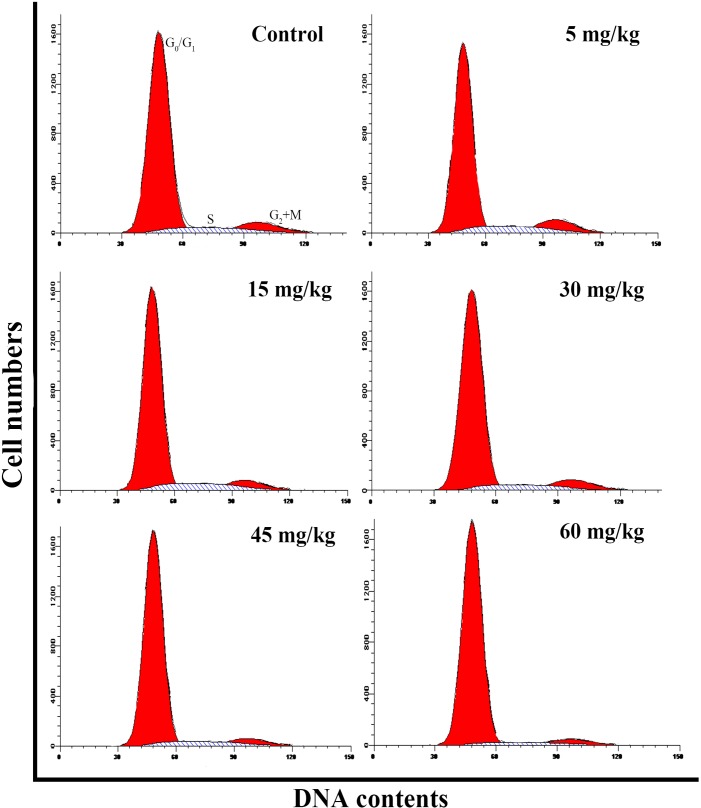
Changes of cell cycle in the thymus by flow cytometry at 42 days of age

**Figure 3:**
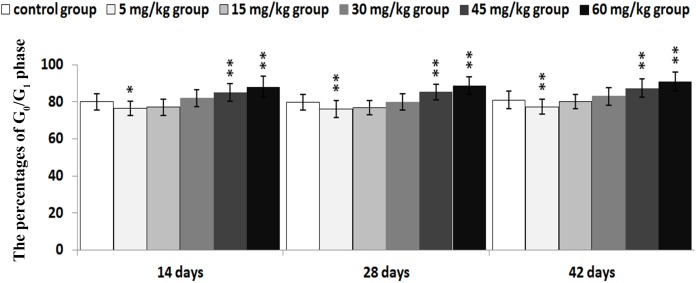
Changes of the percentages of G0/G1 phase in the thymus Data are the means ± standard deviation (n=5) *p<0.05, compared with the control group **p<0.01, compared with the control group.

**Figure 4:**
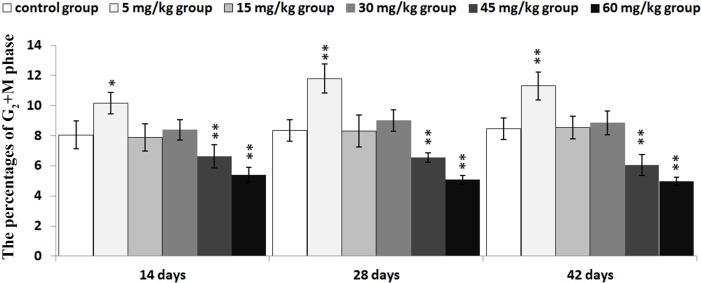
Changes of the percentages of G2+M phase in the thymus Data are the means ± standard deviation (n=5) *p<0.05, compared with the control group **p<0.01, compared with the control group.

**Figure 5:**
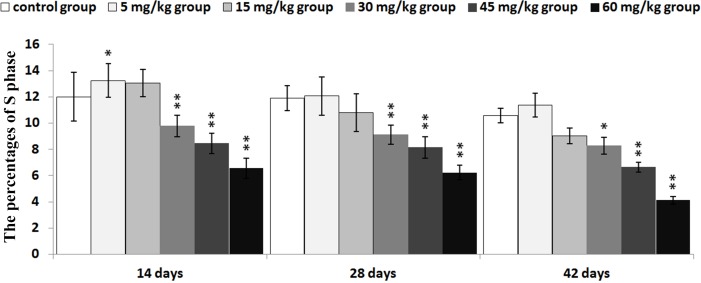
Changes of the percentages of S phase in the thymus Data are the means ± standard deviation (n=5) *p<0.05, compared with the control group **p<0.01, compared with the control group.

**Figure 6:**
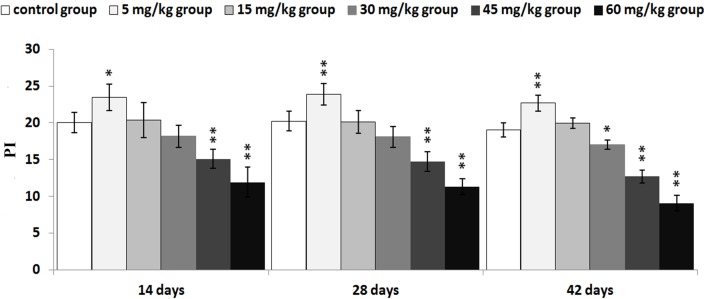
Changes of thymocyte PI (proliferating index) PI =(S+(G2+M))/((G0/G1)+S+(G2+M)) Data are the means ± standard deviation (n=5) *p<0.05, compared with the control group **p<0.01, compared with the control group.

The abovementioned results are shown in Figures 2, 3, 4, 5 and 6.

### Changes of apoptosis in the thymus

#### Flow cytometry method

**Figure 7:**
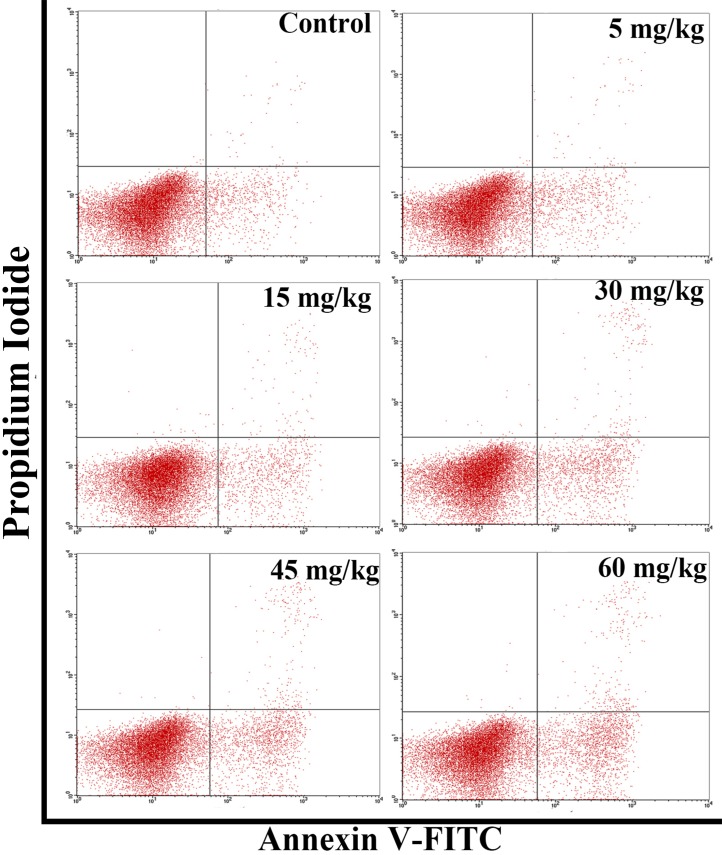
Changes of the apoptosis in the thymus by flow cytometry at 42 days of age

**Figure 8:**
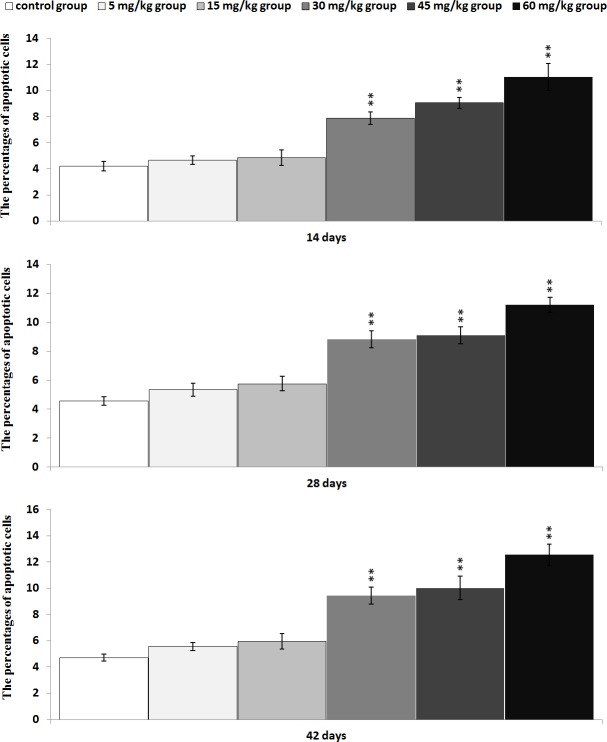
Changes of the percentages of apoptotic cells in the thymus by flow cytometry Data are the means ± standard deviation (n=5) *p<0.05, compared with the control group **p<0.01, compared with the control group.

The results in Figures 7 and 8 showed that the percentages of apoptotic cells in the thymus were increased as dietary vanadium level increased. The percentages of apoptotic thymocytes were significantly higher (*P* < 0.01) in the 30 mg/kg, 45 mg/kg and 60 mg/kg groups than those in the control group.

#### TUNEL assay

**Figure 9:**
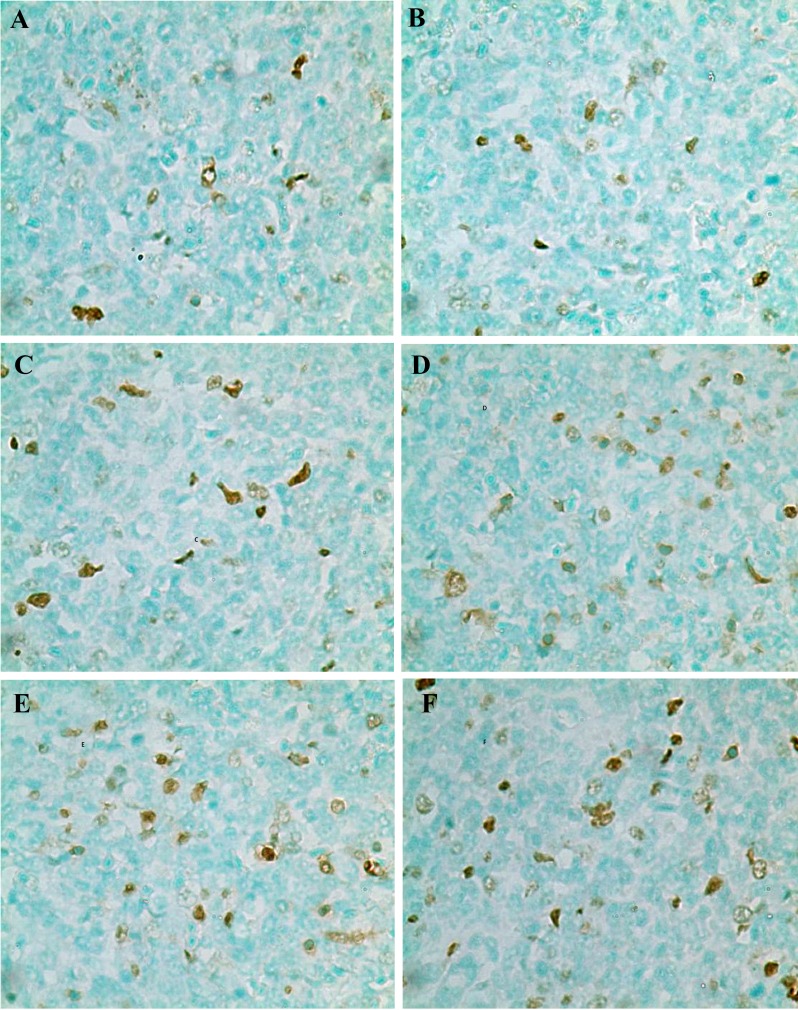
Apoptotic cells in the thymus by TUNEL at 42 days of age There are several apoptotic thymocytes (brown-stained nuclei) in control group **(A)**. The numbers of apoptotic thymocytes are obviously increased in the 30 mg/kg group (**D**), 45 mg/kg group (**E**) and 60 mg/kg group (**F**) at 42 days of age. TUNEL ×1000.

TUNEL assay showed apoptotic thymocytes with brown-stained nuclei. There were increased frequencies of apoptotic thymocytes in the 30 mg/kg, 45 mg/kg and 60 mg/kg groups when compared with those in the control group, as shown in Figure 9.

### Changes of the Bcl-2, Bax and caspase-3 protein expression in the thymus

#### Bax protein expression

**Figure 10:**
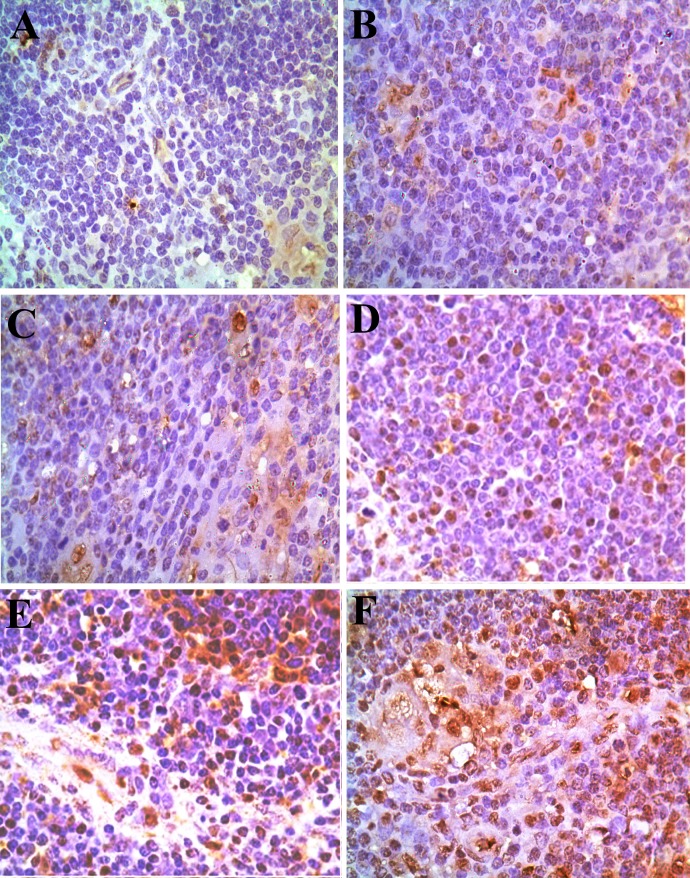
Changes of Bax protein expression in the thymus at 42 days of age There is less Bax protein expression (brown-stained) in control group **(A)**. The Bax protein expression is obviously increased in the 30 mg/kg group (**D**), 45 mg/kg group (**E**) and 60 mg/kg group (**F**) at 42 days of age. Immunohistochemistry ×1000.

**Figure 11:**
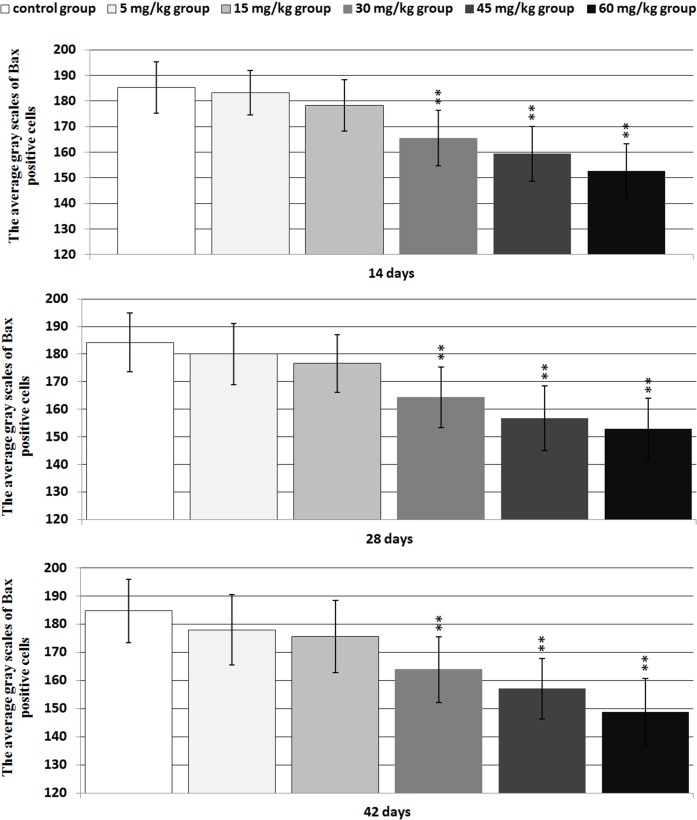
The average gray scales of positive cells containing Bax protein expression in the thymus Immunoreactive intensity was expressed by average grayscale. Values < 160 were considered high expression, 160-170 medium expression and 170-180 low expression. Data are the means ± standard deviation (n=5×5) *p<0.05, compared with the control group **p<0.01, compared with the control group.

Changes of the Bax protein expression levels were no observed in the 5-mg/kg group. The positive thymocytes containing Bax protein expression were increased in the 30mg/kg, 45 mg/kg and 60 mg/kg groups when compared with those of the control group from 14 to 42 days of age. The results were shown in Figures 10 and 11.

#### Bcl-2 protein expression

**Figure 12:**
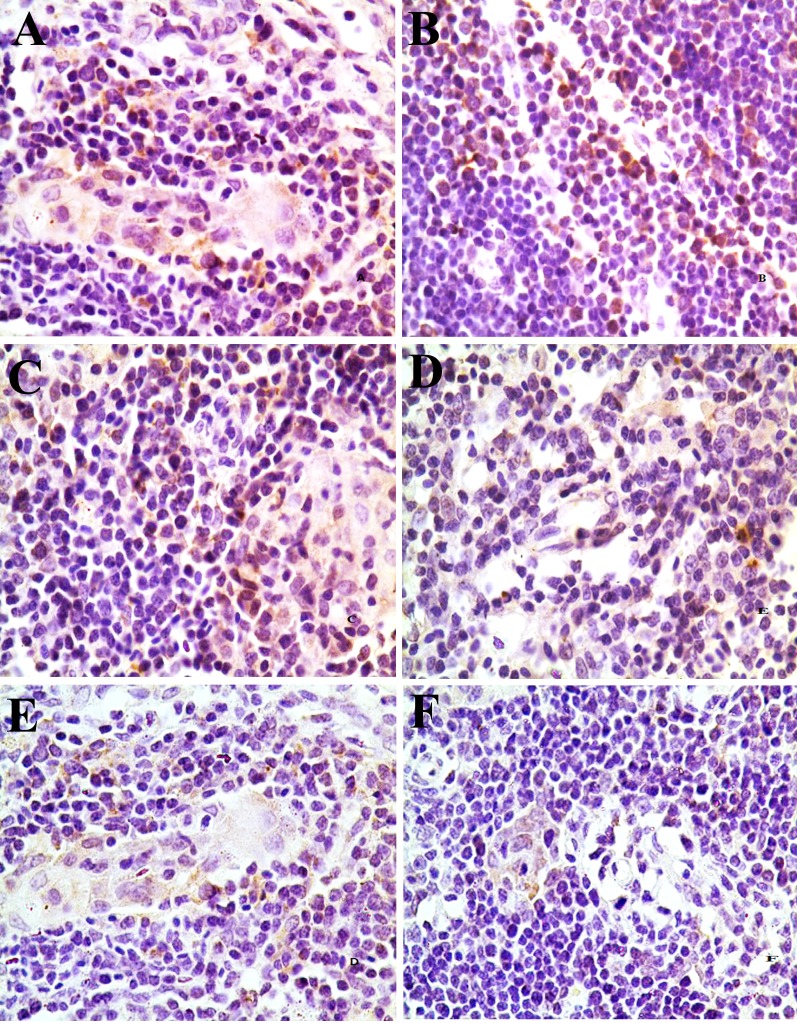
Changes of Bcl-2 protein expression in the thymus at 42 days of age There is abundant Bcl-2 protein expression (brown-stained) in control group **(A)**. The Bcl-2 protein expression is obviously decreased in the 30 mg/kg group (**D**), 45 mg/kg group (**E**) and 60 mg/kg group (**F**) at 42 days of age. Immunohistochemistry ×1000.

**Figure 13:**
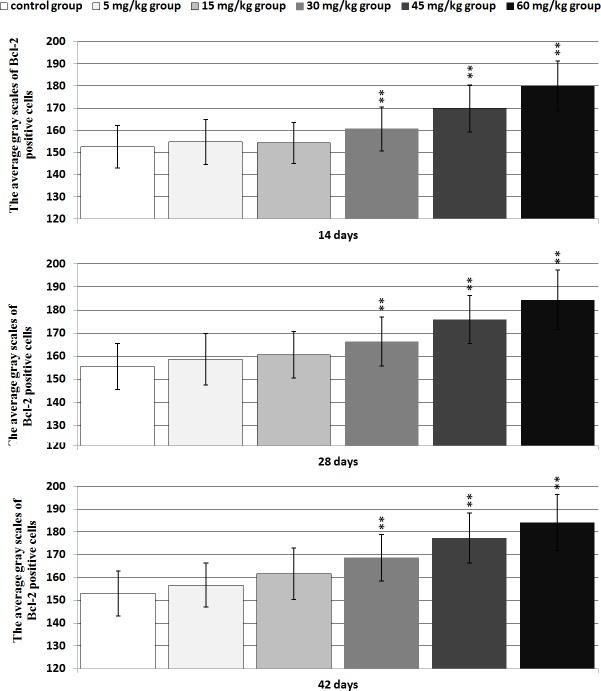
The average gray scales of positive cells containing Bcl-2 protein expression in the thymus Immunoreactive intensity was expressed by average grayscale. Values < 160 were considered high expression, 160-170 medium expression and 170-180 low expression. Data are the means ± standard deviation (n=5×5) *p<0.05, compared with the control group **p<0.01, compared with the control group.

Changes of the Bcl-2 protein expression levels were no observed in the 5-mg/kg group. The positive thymocytes containing Bcl-2 protein expression were lower in the 30mg/kg, 45 mg/kg and 60 mg/kg groups than those of the control group from 14 to 42 days of age. The results were shown in Figures 12 and 13.

#### Caspase-3 protein expression

**Figure 14:**
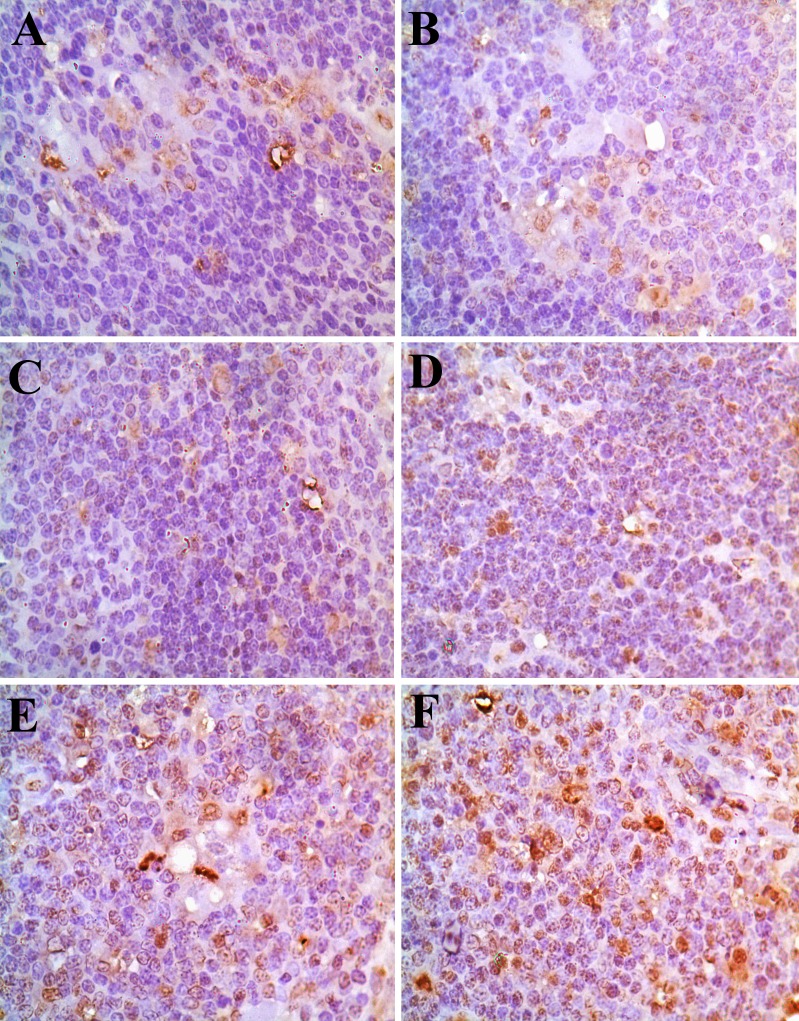
Changes of Caspase 3 protein expression in the thymus at 42 days of age There is less Caspase 3 protein expression (brown-stained) in control group **(A)**. The Caspase 3 protein expression is obviously increased in the 30 mg/kg group (**D**), 45 mg/kg group (**E**) and 60 mg/kg group (**F**) at 42 days of age. Immunohistochemistry ×1000.

**Figure 15:**
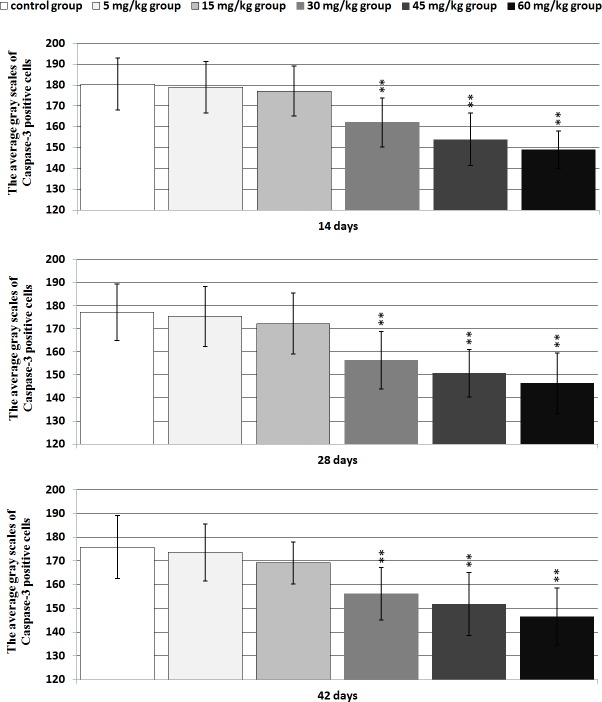
The average gray scales of positive cells containing caspase-3 protein expression in the thymus Immunoreactive intensity was expressed by average grayscale. Values < 160 were considered high expression, 160-170 medium expression and 170-180 low expression. Data are the means ± standard deviation (n=5×5) *p<0.05, compared with the control group **p<0.01, compared with the control group.

Changes of the caspase-3 protein expression levels were consistent with changes of the Bax protein expression levels, as shown in Figures 14 and 15.

### Changes of vanadium contents in the thymus and serum

**Figure 16:**
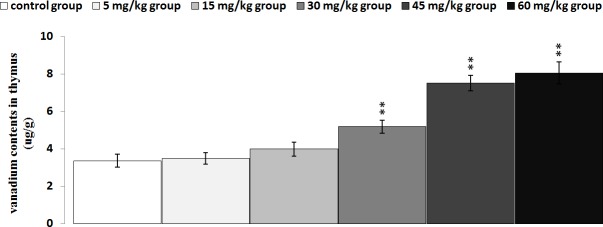
Changes of vanadium contents in the thymus at 42 days Data are the means ± standard deviation (n=5) *p<0.05, compared with the control group **p<0.01, compared with the control group.

**Figure 17:**
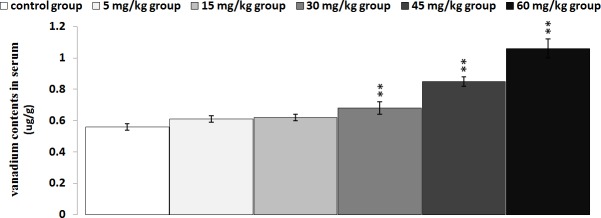
Changes of vanadium contents in the serum at 42 days Data are the means ± standard deviation (n=5) *p<0.05, compared with the control group **p<0.01, compared with the control group.

The results in Figures 16 and 17 showed that the vanadium contents in the thymus and serum were increased as dietary vanadium increased. Vanadium contents were found to be significantly higher (*P* < 0.01 or *P* < 0.05) in the 30 mg/kg, 45 mg/kg and 60mg/kg groups than those in the control group.

## DISCUSSION

The aim of this study was to define the toxic effect of dietary vanadium on thymic development. Indeed, we found considerable evidence that dietary high vanadium ( in the range of 30 mg/kg to 60mg/kg ) had adverse effects on thymic development: reduced relative weight (Figure 1), arrested cell-cycle (Figures 2–6) and increased apoptosis (Figures 7–9). Relative weight has been considered as a satisfactory measure of nutritive value, which can also represent the development state of organs [76]. In the present study, the results showed that the thymic development was obviously inhibited in the three high vanadium groups, which was consistent with the cell cycle arrest.

In order to reveal the mechanism of thymic development suppression caused by dietary high vanadium, we used flow cytometry to analyze the cell cycle of thymocytes. Cell cycle includes S (DNA replication), M (nuclear division and cell division), G_1_ (the cell-cycle gap phase between M phase and S phase), G_2_ (the cell-cycle gap phase between S phase and M phase) phases, which is central to maintain homeostasis in the multicellular organisms [77]. The results showed that high vanadium caused arrest at the G_0_/G_1_ phases, which inhibited damaged cells to stop DNA replication at G_1_ phase, and ultimately resulted in the thymocyte apoptosis when damaged cells can't be repaired. This observation was supported by the findings that high vanadium caused decreased numbers of thymocytes in the S phase and diminished PI of thymocytes (Figures 5 and 6), and increased percentage of apoptotic thymocytes (Figures 7–9). It is clear that the cell cycle arrest and apoptosis of thymocytes can be the main mechanism of thymic development suppression caused by dietary high vanadium.

Apoptosis is the programmed cell death in eukaryotes, which is essential for development and tissue homeostasis by providing a protective mechanism to clean out aged or damaged cells [78, 79]. Apoptosis is tightly controlled by changes, interactions and post-translational modifications (including proteolytic cleavage and phosphorylation) of proteins [80]. In the present study, the results of FCM and TUNEL assays showed that dietary high vanadium increased the percentage of splenocyte apoptosis, which was consistent with the increased Bax and caspase-3 protein expression levels and decreased Bcl-2 protein expression levels in the thymus due to the close relationship between apoptosis and apoptotic protein expression levels. It is well known that increase in pro-apoptotic protein expression or decrease in anti-apoptotic protein expression can push cells down the apoptotic pathway. The Bcl-2 family consists of the anti-apoptotic proteins (Bcl-2-like proteins such as Bcl-2 and Bcl-X_L_), and the pro-apoptotic proteins (such as bax-like and the BH3-only proteins) [81]. Activation of the pro-apoptotic proteins induces cytochrome C release from mitochondria into the cytoplasm [82–85]. Nevertheless, Bcl-2-like proteins can prevent Bax-induced cell death by blocking cytochrome C release [85]. As a pro-apoptotic protein, caspase-3 is activated in the apoptotic cell both by extrinsic (death ligand) and intrinsic (mitochondrial) pathways [86, 87], and executes the apoptotic process [88]. Also, the increased apoptotic thymocytes are closely correlated to the cell cycle arrest caused by dietary high vanadium.

The thymus is essential to the differentiation and development of T cells. T cells develop until mature in the thymus, and then migrate to the peripheral blood and lymphatic system. The thymic development suppression caused by dietary high vanadium can reduce the thymocyte numbers and inhibit the thymic hormone synthesis, implying that mature T lymphocytes in the thymus, and T lymphocytes in the peripheral blood decrease in number [55]. Simultaneously, dietary high vanadium has been reported to reduce T lymphocyte numbers in the lymphatic organ or tissue, such as spleen, cecal tonsil and ileum [57, 60, 64]. T lymphocytes take part in cellular immunity. Thus, the cellular immune function is finally impaired due to the decreased T-lymphocyte numbers and reduced T lymphocyte activities in the peripheral blood and lymphatic organ or tissue caused by dietary high vanadium.

**Figure 18:**
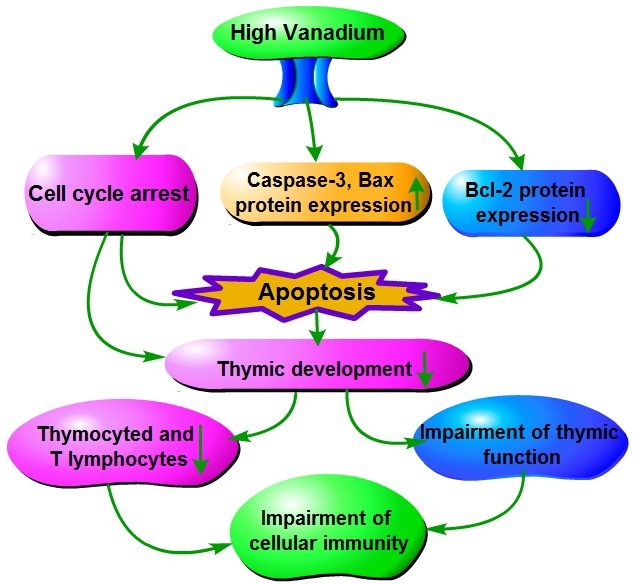
The toxic effect mechanism of vanadium on thymic development Vanadium promotes cell-cycle arrest and caspase-3 and Bax protein expression, and inhibits Bcl-2 protein expression. Cell-cycle arrest, Bax and caspase-3 protein expression up-regulation, and Bcl-2 protein expression down-regulation cause apoptosis. Cell-cycle arrest, apoptosis inhibit thymic development, which finally results in impairment of cellular immunity.

Based on the results of our study and the abovementioned discussion, the toxic effect mechanism of dietary high vanadium on thymic development is summarized in Figure 18.

The decreased relative weight, cell cycle arrest and increased apoptosis percentages are consistent with vanadium accumulation in the thymus (Figures 16 and 17), indicating that the vanadium accumulation is main or/and direct reason of the thymic development suppression.

Concurrently, dietary 5 mg/kg vanadium promotes the thymic development by increasing relative weight, decreasing G_0_/G_1_ phase, increasing S phase and PI, and reducing percentages of apoptotic thymocytes when compared to the control group and high vanadium groups.

In conclusion, dietary high vanadium ( in the range of 30 mg/kg to 60mg/kg ) causes the toxic effect on thymic development by decreasing relative weight, arresting cell cycle, increasing apoptosis percentage. The thymic development suppression caused by dietary high vanadium further leads to inhibitive effects on T lymphocyte maturity and activity, and cellular immune function in the chicken. This study provides a new evidence for further understanding the vanadium immunotoxicity. In contrast, dietary 5 mg/kg vanadium promotes the thymic development by increasing relative weight, decreasing G_0_/G_1_ phase, increasing S phase and PI, and reducing percentages of apoptotic thymocytes when compared to the control group and high vanadium groups.

## MATERIALS AND METHODS

### Chickens and diets

Four hundred and twenty one-day-old healthy broilers were divided into six groups. There were 70 broilers in each group. The broilers were housed in cages with electrically heated units and were provided with water as well as the under-mentioned control or experimental diets *ad libitum* for 42 days.

A corn-soybean basal diet formulated by the National Research Council [89] was the control diet (vanadium 0.073 mg/kg). Ammonium metavanadate (NH_4_VO_3_) was mixed into the corn-soybean basal diet to produce experimental diets with 5 mg/kg, 15 mg/kg, 30 mg/kg, 45 mg/kg and 60 mg/kg of vanadium, respectively.

Our experiments involving the use of broilers and all experimental procedures involving animals were approved by Animal Care and Use Committee, Sichuan Agricultural University.

### Clinical signs and the relative weight of thymus

Clinical signs were observed and recorded every day. At 7, 14, 21, 28, 35 and 42 days of age during the experiment, five broilers in each group were humanely euthanized after they were weighed. Thymus was taken from each broiler and weighed after dissecting connective tissue around the organ. Related weight of thymus was calculated by the following formula:

Related weight = organ weight (g)/body weight (kg)

### Determination of cell cycle stages by flow cytometry

At 14, 28, and 42 days of age, five broilers in each group were selected for determination of the cell-cycle stages in the thymus by flow cytometry, as described by Cui et al. [58].

Thymuses were immediately removed and macerated by grinding to form a cell suspension that was filtered through 300-mesh nylon screen. The cells were washed twice with cold phosphate buffer solution (pH 7.2-7.4), and were then suspended in 1× binding buffer (Cat. No. 51-66121E) at a concentration of 1×10^6^ cells/mL. Five hundred microliters of the solution was transferred to a 5-mL culture tube and centrifuged (500-1,000 rpm). After removing the supernatant, 5μL 0.25% Triton X-100 and 5μL PI (Cat. No. 51-66211E) were added. The cells were gently vortexed and incubated for 30 min at 25°C in the dark. Finally, 500 μL PBS was added to each tube, and the contents were analyzed by flow cytometry (BD FACSCalibur) within 45 min.

### Determination of apoptosis in the thymus

#### Flow cytometry method

At 14, 28, and 42 days of age, five chickens in each group were humanely euthanized. Thymuses were taken for the determination of apoptotic thymocytes by flow cytometry, as described by Peng et al. [90].

The cell suspension was prepared as described in the method of cell of cycle. One hundred microliters of the solution was transferred to a 5-ml culture tube, and then 5 μl of Annexin V-FITC (Cat. No: 51-65874X) and 5 μl of PI (Cat. No: 51-66211E) were added. The cells were gently vortexed and incubated for 15 min at room temperature (25°C) in the dark. Four hundred microliters of 1×binding buffer was added to each tube and analyzed by flow cytometry within 1 h.

#### TUNEL assay

Five broilers in each group were humanely euthanized at 42 days of age. Thymuses were taken and fixed in 10% neutral buffered formalin after postmortem examination, and then processed and trimmed, embedded in paraffin.

TUNEL assay was carried out according to the manual of In Situ Cell Death Detection Kit (Cat: 11684817980, Roche, Germany). Briefly, tissue sections (5 μm thick) were rehydrated in a series of xylene and ethanol solutions and then rinsed in ddH_2_O, digested with proteinase K 50 μl (Tris·HCl pH7.8 diluted) for 15 min, then incubated with 3% H_2_O_2_ in methanol for 15 min at RT (room temperature) to inactivate endogenous peroxidase. Subsequently, sections were transferred to a reaction mixture containing biotin-dUTP terminal deoxynucleotidyl and incubated in a humid chamber for 1 h at 37°C, followed by washing in phosphate buffer saline (PBS, pH 7.2-7.4). Sections were incubated in Converter-POD (HRP) for 30 min at 37°C. Reaction product was visualized with DAB kit (*AR1022, Boster, Wuhan, China).* After a final washes in ddH_2_O, slices were lightly counterstained with hematoxylin, dehydrated in ethanol, cleared in xylene and mounted.

### Determination of Bcl-2, Bax, and caspase-3 protein expression in the thymus by immunohistochemistry

At 14, 28 and 42 days of age, five broilers in each group were humanely euthanized. Thymuses were taken for the detection of Bax, Bcl-2 and caspase-3 protein expression by the immunohistochemical methods (SABC) and stained with DAB as described by Wang et al [91]. Anti-Bax (BA0315), anti-Bcl-2 (BA0412) and anti-caspase-3 (BA0588), and DAB were purchased from Wuhan Boster Biological Technology Co., Ltd., China.

Images from five slices per thymus were taken 200 μm apart. Five visions per slice were randomly chosen for assessment of positive cells using image analysis software (JID801D). The average grayscale of the positive cells was automatically calculated. Immunoreactive intensity was expressed by average grayscale. Values < 160 was considered high, 160-170 medium and 170-180 low.

### Determination of vanadium contents in the thymus and serum

Vanadium contents in the thymus and serum were determined by inductively coupled plasma optical emission spectrometry (ICP-OES).

#### Thymus

At 42 days of age, five thymus in each group were taken and torrefied. 0.1g torrefied sample from each thymus was treated with 2 ml HNO_3_ and 1 ml H_2_O_2_ and dissolved using the automatic microwave-heated digestion system. The volume of the digestive production was increased to 25 ml by the addition of deionized water, which resulted in a clear, colorless solution. At the same time, the blanks of the reagents were created via the same procedure without thymic sample. Then, the treated thymic samples were analyzed by ICP-OES (5,300 V, PE Ltd, USA).

#### Serum

The serum of five chickens in each group was taken at 42 days of age. 1mL serum from each chicken was treated with 2 ml HNO_3_ and 1 ml H_2_O_2_ and dissolved using the automatic microwave-heated digestion system. The volume of the digestive production was increased to 25 ml by the addition of deionized water, which resulted in a clear, colorless solution. At the same time, the blanks of the reagents were created via the same procedure without serum sample. Then, the treated serum samples were analyzed by ICP-OES (5,300 V, PE Ltd, USA).

### Statistical analysis

The significance of difference between the control groups and the vanadium-treated groups was analyzed by the use of variance analysis, and the results are presented as means±standard deviation (X±SD). The analysis was performed with the one-way analysis of variance (ANOVA) test of SPSS 16.0 for windows. A value of *P* < 0.05 was considered significant.
